# Caregiving burden and mental health problems among family caregivers of people with dementia in rural Uganda

**DOI:** 10.1017/gmh.2020.7

**Published:** 2020-05-26

**Authors:** Herbert E. Ainamani, Paul E. Alele, Godfrey Z. Rukundo, Samuel Maling, Edith K. Wakida, Celestino Obua, Alexander C. Tsai

**Affiliations:** 1 Mbarara University of Science and Technology, Mbarara, Uganda; 2 Bishop Stuart University, Mbarara, Uganda; 3 Kabale University School of Medicine, Kabale, Uganda; 4 Center for Global Health and Mongan Institute, Massachusetts General Hospital, Boston, USA; 5 Harvard Medical School, Boston, USA

**Keywords:** Alzheimer's disease, anxiety, caregiving burden, dementia, depression, sub-Saharan Africa, Uganda

## Abstract

**Background:**

Alzheimer's disease and related dementias are associated with increasing health burden in low- and middle-income countries. Less well-recognized is the potential health burden experienced by other affected individuals, such as family caregivers. In this study, we sought to profile the burden of care and its association with symptoms of depression and anxiety among informal caregivers of people living with dementia in rural southwestern Uganda.

**Method:**

We conducted a cross-sectional study of 232 family caregivers of people with dementia. The key measured variables of interest were caregiving burden (Zarit Burden Index) and symptoms of depression and anxiety (Depression Anxiety Stress Scales). We fitted multivariable regression models specifying depression and anxiety symptoms as the primary outcomes of interest and caregiving burden as the primary explanatory variable of interest.

**Results:**

Family caregivers of people with dementia experience significant caregiving burden, with each item on the Zarit Burden Index endorsed by more than 70% of study participants. Nearly half [108 (47%)] of caregivers had Zarit Burden Interview scores >60, suggestive of severe caregiving burden. In multivariable regression models, we estimated a statistically significant positive association between caregiving burden and symptoms of both depression [*b* = 0.42; 95% confidence interval (CI) 0.34–0.49] and anxiety (*b* = 0.37; 95% CI 0.30–0.45).

**Conclusion:**

Family caregivers of people with dementia in rural Uganda experience a high caregiving burden, which is associated with symptoms of depression and anxiety. Interventions aimed at reducing caregiving burden may have important collateral mental health benefits.

## Introduction

The global burden of disease attributable to Alzheimer's disease and related dementias (ADRD) is increasing, placing considerable demands on caregivers (Etters *et al*., 2008). In 2005, 24.3 million people globally were estimated to have dementia, with the majority of cases in low- and middle-income countries (LAMICs) (Ferri *et al*., 2005). The number of people with dementia is projected to increase to 115.4 million people globally by 2050 (Prince *et al*., 2013). Health systems will experience a corresponding rise in global costs of informal and formal care, with total costs worldwide estimated at $818 billion in 2015 (Wimo *et al*., 2017). Across sub-Saharan Africa, the responsibilities of caring for people with ADRD are principally borne by family members (Werner *et al*., 2012; Schatz and Seeley, 2015). It is therefore not surprising that in the 10/66 Dementia Research Group study of caregiving for people with dementia, the median number of hours spent assisting with activities of daily living (ADLs) was highest among caregivers in Nigeria compared with any of the other study sites (Prince and 10/66 Dementia Research Group, 2004). Other studies have identified different aspects of caregiving burden associated with ADRD, including: family caregivers not having enough time for themselves, feeling that their relatives are dependent on them, feelings of embarrassment about the behavior of loved ones with ADRD, feelings of uncertainty about what to do, and many others (Alonso Babarro *et al*., 2004).

A related line of research indicates that the burden of caregiving has been consistently shown to have adverse effects on emotional wellbeing, but most of these studies have been derived from high-income countries (Pinquart and Sorensen, 2003; Ferrara *et al*., 2008). Moreover, exacerbating this caregiving burden in LAMICs are the generally low levels of awareness about dementia as a neurodegenerative disease, poor access to high-quality health care services for people with dementia, stigma attached to dementia and cognitive impairment, and lack of adequate funding for education/training and program implementation (de Jager *et al*., 2017; Johnston *et al*., 2019). These findings are supported by recent studies from Uganda and Tanzania that observed low levels of knowledge about dementia among clinicians and within the general population (Mushi *et al*., 2014; Kamoga *et al*., 2019). Results of studies on caregiving burden and mental health problems among caregivers of people living with dementia from sub-Saharan Africa, however, have been mixed. One study from Kenya showed that caregivers had poorer mental health compared with non-caregivers (Ice *et al*., 2010), but the investigators did not measure caregiving burden to assess the range of caregiving burden severity within the sample of caregivers. Other studies have identified stressors associated with caregiving for other types of dependents, including people with advanced cancer, HIV, and schizophrenia (Kipp *et al*., 2006; Ukpong, 2006). And finally, a related study from Tanzania compared caregiving burden among caregivers of older-age persons and persons with Parkinson's disease or dementia but did not measure its association with mental health problems (Dotchin *et al*., 2014). In that study, the authors observed that caregivers of people with dementia experienced less caregiving burden compared with caregivers of older-age persons and persons with (non-Parkinson's) dementia. We sought to address this gap in the literature by conducting a cross-sectional study in rural Uganda to estimate the association between caregiving burden and mental health among family caregivers of people with dementia.

## Method

### Study setting

In this cross-sectional study, we used structured interviews to collect survey data from 232 family caregivers of patients with dementia who were in care at selected health centers in the Rukiga and Rubanda districts of southwestern Uganda. The largest town in the region is Kabale, with a population of approximately 50000. Most residents of these districts live in outlying rural areas, where the local economy is primarily driven by subsistence agriculture, animal husbandry, and petty trading. Food and water insecurity are common (Tsai *et al*., 2012, 2016; Mushavi *et al*., 2020).

### Recruitment and sampling procedure

The study was based at two health centers, Reach One Touch One Ministries (Rukiga) and Heal Medical Centre (Rubanda). Using convenience sampling, we identified adult caregivers of people with dementia who lived in the same home or compound with their corresponding patients and who provided care for at least 6 months. Caregivers who could not communicate effectively with research staff (e.g. due to deafness/mutism, acute intoxication, or other cognitive impairment as determined in the field with supervision by a clinical psychologist). Survey instruments were written in English, translated into the local language (Rukiga-Runyankore), and back-translated to ensure fidelity to the original text. They were then administered by the principle investigator and two interviewers who received 2 weeks of training on how to collect sensitive data and screen for mental health problems. Interviews were conducted in a private setting, in either English or Rukiga-Runyankore, depending on caregivers' preferences. Each participant provided oral and written informed consent. If a signature could not be obtained for literacy reasons, participants were permitted to indicate consent with a fingerprint. At the end of the interview, consistent with local etiquette, each participant received a bar of soap and a package of salt for their participation in the study.

### Ethical considerations

Ethical clearance to conduct this research study was obtained from the Mbarara University of Science and Technology Research Ethics Committee. Consistent with national guidelines, we also obtained clearance for the study from the Uganda National Council for Science and Technology and the Research Secretariat in the President's Office.

### Variables of interest

The primary outcomes of interest were caregivers' symptoms of depression and anxiety, which we assessed using the depression and anxiety subscales of the 42-item Depression Anxiety and Stress Scales (DASS) (Lovibond and Lovibond, 1995). Each subscale consists of 14 items scored on a 4-point Likert-type scale ranging from ‘never’ to ‘almost always.’ Scores range from 0 to 42, with higher scores reflecting endorsement of more symptoms of anxiety or depression. The DASS shows adequate internal consistency, with evidence of construct validity suggested by robust correlations with theoretically associated constructs (Crawford and Henry, 2003; Gloster *et al*., 2008). In the current study, the Cronbach's *α* was 0.89.

Caregiving burden was assessed using the 22-item Zarit Burden Interview (ZBI) (Zarit *et al*., 1980). The ZBI contains 22 items eliciting different aspects of caregiving burden, each scored on a 5-point Likert scale ranging from ‘never’ to ‘nearly always.’ Scores range from 0 to 88, with higher scores reflective of greater caregiving burden. Following the guidance by Zarit and Zarit (1987), ZBI scores were used to categorize participants into four categories of increasing caregiving burden: low (0–20), moderate (21–40), high (41–60), and severe (61–88). The ZBI has previously been used to characterize caregiving burden in many high-income settings, including the USA, Japan, and Europe (Edwards and Scheetz, 2002; Liew *et al*., 2018). The ZBI has been less frequently used to assess the caregiving burden in sub-Saharan Africa (Dotchin *et al*., 2014; Kidman and Thurman, 2014; Imarhiagbe *et al*., 2017). In the current study, Cronbach's *α* was 0.91.

In addition to the primary caregiver variables of interest, we collected information on caregivers' age, sex, education, and duration of involvement with providing care. We also collected information on the people with dementia being cared for by the caregivers in our study (hereafter: ‘index patient’). To assess functional impairment, we administered the caregiver-report Bristol Activities of Daily Living Scale (BADLS) (Bucks *et al*., 1996). BADLS scores were used to assess the severity of functional impairment on a continuous scale ranging from 0 to 60. While there are no validated scoring thresholds, some have proposed scoring categories that range from ‘functionally independent’ (BADLS = 0) to significant functional impairment (BADLS range, 8–36) and severe functional impairment (BADLS >36) (Brefka *et al*., 2019). Finally, we also recorded the age and sex of the index patient and the index patient's relationship with the caregiver.

### Data analysis

Statistical analyses were performed in SPSS Statistics 22 for Mac (SPSS Inc., Chicago, IL). First, the DASS subscales and ZBI were specified as continuous variables. Then the ZBI was specified as a categorical variable using the cut-offs described above. For each outcome, the first regression model included only the ZBI. The second regression model also adjusted for potential confounding by caregivers' age, sex, education, and years of care; and by age and sex of the index patient, and the index patient's relationship with the caregiver.

### Sensitivity analysis

This study used a cross-sectional, observational design; therefore, confounding by unmeasured covariates could potentially explain the observed associations between caregiving burden and mental health outcomes. To quantify the extent to which unmeasured confounding could have explained our findings, we computed the e-value as defined by VanderWeele and Ding (2017): the e-value is defined as the minimum strength of association, on the risk ratio scale, that an unmeasured covariate would need to have with both the exposure (caregiving burden) and outcome (mental health) in order to render the estimated association null.

Owing to skewness in the severity of the ZBI scores, we conducted two sensitivity analyses for the categorical specification using different cutoff scores. First, we used the ZBI score cutoffs identified in a population-based study of community-dwelling persons with dementia in Canada (Hébert *et al*., 2000): low, 0–8; moderate, 9–17; high, 18–32; and severe, 33–88. Second, we used our data to define deciles of the ZBI: first decile (lowest), 4–34; second decile, 35–44; third decile, 45–49; fourth decile, 50–55; fifth decile, 56–59; sixth decile, 60–63; seventh decile, 64–67; eighth decile, 68–74; ninth decile, 75–80; tenth decile (highest), 81–88.

## Results

### Characteristics of the sample

All study participants were enrolled and interviewed in December 2018 and January 2019. The sample included 232 family caregivers who stayed in the same house or compound as the index patient and who had provided care for more than 6 months (Table 1). Most caregivers were women [141 (61%)]. Among women caregivers, the mean age was 44 years (s.d., 15.4), and they provided caregiving for a mean duration of 5.4 years (s.d., 3.3). The mean DASS depression subscale score was 25.0 (s.d., 12.2) for women and 21.5 (s.d., 11.8) for men (*t* = 2.11 for comparison; *p* = 0.036). The mean DASS anxiety subscale score was 24.5 (s.d., 11.4) for women and 19.9 (s.d., 11.4) for men (*t* = 3.00 for comparison; *p* = 0.003). Mean ZBI scores were slightly higher among women compared with men, but the difference was not statistically significant (58.9 *v*. 55.0; *t* = 1.68 for comparison; *p* = 0.09). Among men caregivers, only four (4.4%) were categorized as having ‘low’ caregiving burden while 15 (16%) had ‘moderate’ caregiving burden, 33 (36%) had ‘high’ caregiving burden, and 39 (43%) had ‘severe’ caregiving burden. Among women caregivers, only three (2.1%) were categorized as having ‘low’ caregiving burden, while 14 (9.9%) had ‘moderate’ caregiving burden, 55 (39%) had ‘high’ caregiving burden, and 69 (49%) had ‘severe’ caregiving burden.

**Table 1.** Characteristics of the caregiver sample (*N* = 232)

	Men (*N* = 91)		Women (*N* = 141)			
	Mean or *N* (%)	s.d.	Mean or *N* (%)	s.d.	*t* or χ^2^	*p* value
Age, years	46.3	16.2	43.8	15.4	1.19	0.23
Education, years	2.3	0.83	1.8	0.82	4.56	<0.001
Duration of caregiving, years	5.6	4.37	5.4	3.32	0.52	0.60
DASS depression subscale	21.5	11.8	25.0	12.2	−2.11	0.04
DASS anxiety subscale	19.9	11.4	24.5	11.4	−3.00	0.003
BADLS	40.5	10.6	40.5	10.9	−0.02	0.98
ZBI Score	55.0	18.5	58.9	16.0	−1.68	0.09
Low (0–20)	4 (4.4)		3 (2.1)		3.39	0.34
Moderate (21–40)	15 (16)		14 (9.9)			
High (41–60)	33 (36)		55 (39)			
Severe (>61)	39 (43)		69 (49)			

DASS, Depression and Anxiety Stress Scales; ZBI, Zarit Burden Interview.

Among the index patients, there were 40 (17%) men with a mean age of 83.3 years, [standard deviation (s.d.), 10.8], and 192 (83%) women with a mean age of 84 years (s.d., 11.2). The mean BADLS score was 41.7 (s.d., 11.6) for men and 42.1 (s.d., 11.1) for women.

### Caregiving burden

Almost all participants in our sample endorsed at least one aspect of caregiving burden (Table 2). The most frequently endorsed items were: not having enough time for self [230 (99%)], patients being too dependent [229 (99%)], feeling financially stressed [28 (98%)], feeling like they could be doing more for the patient [228 (98%)], and feeling like they should be doing better for the patient [228 (98%)]. More women compared with men reported feeling strained being around the patient, feeling uncertain about what to do, and feeling an inability to take care of the patient (Table 2).

**Table 2.** Endorsement of individual items on the Zarit Burden Interview, stratified by caregiver sex

	All, *N* (%)	Women, *N* (%)	Men, *N* (%)	χ^2^	*p*
Not having enough time for self	230 (99)	141 (100)	89 (98)	3.13	0.08
Patient being too dependent	229 (99)	140 (99)	89 (98)	0.96	0.33
Feeling financially stressed	228 (98)	140 (99)	88 (97)	2.19	0.14
Feel doing more for the patient	228 (98)	139 (99)	89 (98)	0.20	0.66
Feeling of doing better for the patient	228 (98)	139 (99)	89 (98)	0.20	0.66
Overall Burden in caring for a relative	227 (98)	140 (99)	87 (96)	3.56	0.06
Patient asks for more help than he/she needs	227 (98)	137 (97)	90 (99)	0.79	0.37
Being afraid about the patient's future	223 (96)	138 (98)	85 (93)	2.96	0.09
Patient expecting me to be the only caregiver	222 (96)	138 (98)	84 (92)	4.15	0.04
Feeling stressed about fulfilling other responsibilities	221 (95)	136 (96)	85 (93)	1.14	0.29
Feeling strained being around my patient	211 (91)	134 (95)	77 (85)	7.29	0.01
Suffering in social life	209 (90)	132 (94)	77 (85)	5.02	0.03
Feeling embarrassed over patient's behaviors	206 (89)	127 (90)	79 (87)	0.59	0.44
Feeling uncertain of what to do	204 (88)	133 (94)	71 (78)	13.9	<0.001
Suffering from ill health because of caregiving	200 (86)	128 (91)	72 (79)	6.32	0.01
Feeling of inability to take care of my patient very much	195 (84)	128 (91)	67 (74)	12.1	<0.001
Feeling of losing control over my life because of caregiving	195 (84)	125 (89)	70 (77)	5.68	0.02
Having inadequate privacy because of caregiving	189 (82)	123 (87)	66 (73)	7.92	0.01
Feeling angry being around my patient	171 (74)	111 (79)	60 (66)	4.67	0.03
Wishing to leave care of my patient	169 (73)	108 (77)	61 (67)	2.56	0.11
Caring for my patient has negatively affected relationships	168 (72)	108 (77)	60 (66)	3.15	0.07
Feeling uncomfortable having friends visit	168 (72)	107 (76)	61 (67)	2.17	0.14

### Association between caregiving burden and depression symptom severity

In a regression model with just a single explanatory variable, the ZBI had a statistically significant association with depression symptom severity [*b* = 0.42; 95% confidence interval (CI) 0.36–0.48] and explained 35% of the variance in depression symptom severity (Table 3). After adjustment for covariates, the estimated association between caregiving burden and depression symptom severity remained statistically significant (*b* = 0.42; 95% CI 0.34–0.49). This regression model explained 41% of the variance in depression symptom severity. The estimated association was large in magnitude: a one-standard deviation increase in the ZBI (17.0) was associated with a 17 × 0.42 = 7.14 greater depression symptom severity score, which was 7.14/23.6 = 30% of the sample mean and 7.14/12.2 = 0.59 standard deviation units. Other statistically significant predictor variables were the age and sex of the caregiver and age of the index patient.

**Table 3.** Association between caregiving burden and depression symptom severity

	*b*	s.e.	*t*	*p*	95% CI	
*Model 1*						
ZBI, total score	0.42	0.03	13.6	<0.001	0.36	0.48
*Model 2*						
ZBI, total score	0.42	0.04	10.6	<0.001	0.34	0.49
BADLS, total score	−0.04	0.08	−0.55	0.58	−0.19	0.11
Age of caregiver (years)	0.18	0.05	3.31	0.001	0.07	0.28
Sex of caregiver (female)	3.06	1.51	2.03	0.04	0.09	6.03
Education (years)	−0.32	0.93	−0.34	0.73	−2.15	1.51
Duration of care provided (years)	0.20	0.20	1.01	0.32	−0.19	0.58
Age of index patient (years)	0.15	0.07	2.09	0.04	0.01	0.28
Sex of index patient (female)	−1.91	2.12	−0.90	0.37	−6.08	2.27
Relationship of index patient to caregiver						
Parent/parent-in-law	Ref					
Grandparent	2.66	2.06	1.29	0.20	−1.40	6.73
Spouse/other	0.02	2.24	0.01	0.99	−4.41	4.44

*b*, estimated regression coefficient; s.e., standard error; CI, confidence interval; ZBI, Zarit Burden Interview, BADLS, Bristol Activities of Daily Living Scale.

### Association between caregiving burden and anxiety symptom severity

In a regression model with just caregiving burden as the explanatory variable, the ZBI had a statistically significant association with anxiety symptoms (*b* = 0.37; 95% CI 0.32–0.43) (Table 4). This first model explained 30% of the variance in anxiety symptoms. After adjustment for covariates, the estimated association between caregiving burden and anxiety symptoms remained statistically significant (*b* = 0.37; 95% CI 0.30–0.45). This regression model explained 38% of the variance in anxiety symptoms. Other statistically significant predictor variables were the age and sex of the caregiver.

**Table 4.** Association between caregiving burden and anxiety symptom severity

	*b*	s.e.	*t*	*p*	95% CI	
*Model 1*						
ZBI, total score	0.37	0.03	12.38	<0.001	0.32	0.43
*Model 2*						
ZBI, total score	0.37	0.39	9.55	<0.001	0.30	0.45
BADLS, total score	−0.04	0.08	−0.58	0.56	−0.19	0.11
Age of caregiver (years)	0.16	0.05	3.08	<0.01	0.06	0.26
Sex of caregiver (female)	3.78	1.54	2.45	0.02	0.74	6.83
Education (years)	−1.08	1.04	−1.04	0.30	−3.14	0.98
Duration of care provided (years)	0.20	0.17	1.20	0.23	−0.13	0.53
Age of index patient (years)	0.11	0.07	1.68	0.09	−0.02	0.25
Sex of index patient (female)	−2.31	2.03	−1.13	0.26	−6.32	1.71
Relationship of index patient to caregiver						
Parent/parent-in-law	Ref					
Grandparent	2.67	1.94	1.37	0.17	−1.17	6.51
Spouse/other	−0.51	2.36	−0.22	0.83	−5.18	4.15

*b*, estimated regression coefficient; s.e., standard error; CI, confidence interval; ZBI, Zarit Burden Interview, BADLS, Bristol Activities of Daily Living Scale.

### Analysis of caregiving burden as a categorical variable

When the ZBI was specified as a categorical explanatory variable, we found that the different categories of caregiving burden (relative to low caregiving burden) had associations with depression symptom severity that increased across categories: moderate, *b* = 7.8 (95% CI −1.41 to 14.3); high, *b* = 17.6 (95% CI 11.9–23.2); and severe, *b* = 23.5 (95% CI 17.7–29.3). A similar pattern was observed in the associations with anxiety symptoms: moderate, *b* = 9.3 (95% CI 3.7–14.8); high, *b* = 18.7 (95% CI 13.9–23.6); and severe, *b* = 23.5 (95% CI 18.3–28.6) (Appendix Table 1).

### Sensitivity analyses

The estimated associations appeared to be robust to potential confounding by unobserved variables and alternative specifications. In the multivariable linear regression model estimating the association between caregiving burden and depression symptom severity, in order to fully explain away the effect of a one-standard deviation increase in caregiving burden, an unmeasured confounder would need to have a very strong association (relative risk of 2.8) with both caregiving burden and with depression symptom severity. The e-value for the effect of severe caregiving burden on depression, taken from Appendix Table 1, exceeds 10. Thus, any residual confounding would need to be extremely strong in order to explain away our findings.

The category thresholds originally proposed by Zarit and Zarit (1987) have been criticized for their arbitrariness (Hébert *et al*., 2000). In addition, in our data there was a skewed distribution of the scores. For these reasons, we conducted sensitivity analyses to probe the robustness of our findings. Using the score cut-offs provided by Hébert *et al*. (2000), we found that the majority of study participants [212 (91%)] scored in the highest category of severity. Accordingly, in the multivariable regression models using these cut-offs, only the highest-severity category of the ZBI had a statistically significant association with either the DASS depression subscale (*b* = 20.6; 95% CI 13.6–27.5) or the DASS anxiety subscale (*b* = 20.9; 95% CI 14.8–26.9) (Appendix Table 2).

Next, we categorized ZBI scores into deciles and included this categorical variable in the multivariable regression models. In the multivariable regression models alternately specifying the DASS depression and anxiety subscales as the outcomes, all of the decile categories had statistically significant associations with the outcome. The linear tests for trend were statistically significant for both depression and anxiety (*p* < 0.001) (Appendix Table 3).

## Discussion

This cross-sectional study sought to profile caregiving burden and mental health problems among family caregivers of people living with dementia in rural Uganda. We found that overall caregiving burden was high and was principally related to the duties of caregiving, stress, and financial concerns. This high caregiving burden may be partially explained by the undeveloped health systems in rural settings of African countries such as the one explored in this study (a rural region of southwestern Uganda). Our findings are consistent with prior work describing a high caregiving burden among family caregivers for people with dementia.

The second primary contribution of our study was to document a robust association between caregiving burden and mental health problems. The estimated associations were large in magnitude, robust to potential confounding by socio-demographic characteristics, and also unlikely to be explained away by unmeasured covariates. Our findings are consistent with a number of studies that have estimated a correlation between caregiving burden and mental health problems, but most have been conducted in high-income countries. Taken together, these findings lend support to the notion that dementia not only affects cognitive function among people with dementia but also has significant spillover effects on mental health status among family caregivers (Kalaria *et al*., 2008; Brodaty and Donkin, 2009). Thus, programming and policy-oriented toward the prevention of early dementia may serve a dual function: to improve population health and also to lessen caregivers' burden. Previous research, for example, has indicated that green space and participation in gardening activities may be beneficial from the perspective of depression and anxiety symptoms (Barton and Rogerson, 2017; Kam and Siu, 2010). In addition, other studies have shown promising effects of cognitive stimulation not only for older-age people with dementia but also caregiver wellbeing (Mkenda *et al*., 2018; Paddick *et al*., 2017). Our findings are also consistent with recommendations to introduce a core program in LAMICs of early diagnosis and needs assessments for people with ADRD, coupled with informational and social support for caregivers (Prince *et al*., 2009). We argue that further research on the health effects associated with caregiving in LAMICs should be encouraged.

Interpretation of our findings should be limited by several considerations. First, the cross-sectional nature of our study design does not allow us to assess the extent to which caregiving burden causes mental health problems (or whether, for example, people with mental health problems are more likely to experience caregiving burden). Prospective studies are needed to shed light on the causal relations. However, the e-value analysis indicates that residual confounding would need to be extremely strong in order to explain away the findings. Second, the convenience sampling strategy could have enriched the sample for caregivers of people with the early-stage illness. While possible, we believe it to be unlikely, given the large proportion of the sample with BADLS values consistent with severe functional impairment. In addition, there were no refusals. Third, all of the variables were elicited through structured interviews, so it is possible that there could be measurement error and/or confounding related to negative affect. Misclassification of depression or anxiety status could have been differential if people with greater caregiving burden were more likely to be experiencing negative emotions and therefore more likely to over-report symptoms of depression and anxiety – thereby causing bias away from the null.

In summary, our findings indicate that family caregivers of people living with dementia in rural Uganda are highly burdened and experience significant mental distress. Physicians and allied health professionals should consider probing for mental health problems among family caregivers while providing care to their patients with dementia.

## Supplementary material

For supplementary material accompanying this paper visit http://dx.doi.org/10.1017/gmh.2020.7. click here to view supplementary material

## Data

The data for the current study are available from the corresponding author on request.
