# Supplementary material for: Caregiving burden and mental health problems among family caregivers of people with dementia in rural Uganda
Source: Glob Ment Health (Camb). 2020 May 26;7:e13. doi: 10.1017/gmh.2020.7 (PMC7379317; doi:10.1017/gmh.2020.7)
Supplement: Supplementary file 1 [file S2054425120000072sup001.doc]

**Appendix Table 1. Association between caregiving burden categories and depression and anxiety symptom severity, using categories suggested by Zarit & Zarit (1987)**

|  | Depression | | | | Anxiety | | | |
| --- | --- | --- | --- | --- | --- | --- | --- | --- |
|  | b (SE) | p | 95% CI | | b (SE) | p | 95% CI | |
| ZBI categories, Zarit and Zarit (1987) |  |  |  |  |  |  |  |  |
| Low (≤20) | Ref |  |  |  | Ref |  |  |  |
| Moderate (21-40) | 7.8 (3.2) | 0.02 | (1.41, | 14.3) | 9.25 (2.82) | 0.001 | (3.68, | 14.8) |
| High (41-60) | 17.6 (2.8) | <0.001 | (11.9, | 23.2) | 18.7 (2.47) | <0.01 | (13.9, | 23.6) |
| Severe (61-88) | 23.5 (2.9) | <0.001 | (17.7, | 29.3) | 23.5 (2.61) | <0.01 | (18.3, | 28.6) |
| BADLS | 0.01 (0.08) | 0.87 | (-0.14, 0.16) | | -0.01 (0.07) | 0.89 | (-0.15, | 0.13) |
| Age of caregiver (years) | 0.17 (0.06) | <0.01 | (0.06, | 0.29) | 0.16 (0.06) | <0.01 | (0.05, | 0.27) |
| Sex of caregiver (female) | 2.99 (1.61) | 0.07 | (-0.20, | 6.18) | 3.63 (1.60) | 0.02 | (0.48, | 6.79) |
| Education (years) | -0.62 (0.98) | 0.53 | (-2.56, | 1.31) | -1.43 (1.08) | 0.19 | (-3.55, | 0.70) |
| Duration of care provided (years) | 0.24 (0.21) | 0.27 | (-0.18, | 0.66) | 0.25 (0.18) | 0.16 | (-0.10, | 0.60) |
| Age of index patient (years) | 0.14 (0.07) | 0.05 | (0.00, | 0.27) | 0.11 (0.10) | 0.11 | (-0.02, | 0.24) |
| Sex of index patient (female) | -2.65 (2.41) | 0.27 | (-7.40, | 2.10) | -3.00 (2.24) | 0.18 | (-7.42, | 1.43) |
| Relationship of index patient to caregiver |  |  |  |  |  |  |  |  |
| Parent/parent-in-law | Ref |  |  |  | Ref |  |  |  |
| Grandparent | 4.09 (2.25) | 0.07 | (-0.36, | 8.53) | 4.16 (2.07) | 0.05 | (0.08, | 8.25) |
| Spouse/other | -0.67 (-0.27) | 0.79 | (-5.56, | 4.21) | -1.23 (2.54) | 0.63 | (-6.24, | 3.78) |

*b, estimated regression coefficient; SE, standard error; CI, confidence interval; ZBI, Zarit Burden Interview*

**Appendix Table 2. Association between caregiving burden categories and depression and anxiety symptom severity, using categorized derived by Hébert et al. (2000)**

|  | Depression | | | | Anxiety | | | |
| --- | --- | --- | --- | --- | --- | --- | --- | --- |
|  | b (SE) | p | 95% CI | | b (SE) | p | 95% CI | |
| ZBI categories, Hébert et al. (2000) |  |  |  |  |  |  |  |  |
| Low or Moderate (≤17) | Ref |  |  |  | Ref |  |  |  |
| High (18-32) | 3.09 (4.12) | 0.54 | (-5.05, | 11.2) | 3.59 (3.55) | 0.31 | (-3.42, | 10.6) |
| Severe (33-88) | 20.6 (3.54) | <0.001 | (13.6, | 27.5) | 20.9 (3.09) | <0.001 | (14.8, | 26.9) |
| BADLS | 0.07 (0.08) | 0.39 | (-0.09, | 0.23) | 0.04 (0.07) | 0.63 | (-0.11, | 0.18) |
| Age of caregiver (years) | 0.18 (0.06) | 0.003 | (0.06, | 0.30) | 0.17 (0.06) | <0.01 | (0.06, | 0.29) |
| Sex of caregiver (female) | 2.97 (1.65) | 0.07 | (-0.28, | 6.23) | 3.59 (1.62) | 0.03 | (0.40, | 6.79) |
| Education (years) | -0.67 (0.91) | 0.46 | (-2.47, | 1.13) | -1.42 (1.00) | 0.16 | (-3.40, | 0.55) |
| Duration of care provided (years) | 0.18 (0.19) | 0.34 | (-0.19, | 0.56) | 0.19 (0.17) | 0.27 | (-0.15, | 0.52) |
| Age of index patient (years) | 0.13 (0.07) | 0.07 | (-0.01, | 0.27) | 0.10 (0.07) | 0.14 | (-0.03, | 0.24) |
| Sex of index patient (female) | 0.58 (2.38) | 0.80 | (-4.05, | 5.21) | 0.07 (2.19) | 0.98 | (-4.25, | 4.39) |
| Relationship of index patient to caregiver |  |  |  |  |  |  |  |  |
| Parent/parent-in-law | Ref |  |  |  | Ref |  |  |  |
| Grandparent | 4.76 (2.33) | 0.04 | (0.16, | 9.37) | 4.74 (2.14) | 0.03 | (0.51, | 8.96) |
| Spouse/other | 2.27 (2.62) | 0.39 | (-2.91, | 7.45) | 1.48 (2.59) | 0.57 | (-3.62, | 6.58) |

*b, estimated regression coefficient; SE, standard error; CI, confidence interval; ZBI, Zarit Burden Interview*

**Appendix Table 3. Association between caregiving burden categories and depression and anxiety symptom severity, using deciles of the ZBI**

|  | Depression | | | | Anxiety | | | |
| --- | --- | --- | --- | --- | --- | --- | --- | --- |
|  | b (SE) | p | 95% CI | | b (SE) | p | 95% CI | |
| ZBI deciles |  |  |  |  |  |  |  |  |
| 1st (lowest; 4-34) | Ref |  |  |  | Ref |  |  |  |
| 2nd (35-44) | 12.4 (2.82) | <0.001 | (6.81, | 18.0) | 12.3 (2.58) | <0.001 | (7.22, | 17.4) |
| 3rd (45-49) | 18.4 (2.92) | <0.001 | (12.6, | 24.1) | 18.3 (2.86) | <0.001 | (12.6, | 23.9) |
| 4th (50-55) | 15.2 (3.43) | <0.001 | (8.40, | 22.0) | 14.1 (3.61) | <0.001 | (6.91, | 21.2) |
| 5th (56-59) | 15.3 (2.90) | <0.001 | (9.60, | 21.0) | 15.9 (2.71) | <0.001 | (10.6, | 21.3) |
| 6th (60-63) | 18.7 (2.81) | <0.001 | (13.2, | 24.3) | 18.3 (2.76) | <0.001 | (12.8, | 23.7) |
| 7th (64-67) | 16.8 (3.01) | <0.001 | (10.8, | 22.7) | 16.3 (2.73) | <0.001 | (10.9, | 21.7) |
| 8th (68-74) | 26.1 (2.22) | <0.001 | (21.7, | 30.5) | 24.4 (2.04) | <0.001 | (20.4, | 28.4) |
| 9th (75-80) | 23.9 (2.42) | <0.001 | (19.1, | 28.7) | 22.2 (2.27) | <0.001 | (17.7, | 26.7) |
| 10th (highest; 81-88) | 29.4 (2.51) | <0.001 | (24.5, | 34.4) | 25.2 (2.82) | <0.001 | (19.6, | 30.7) |
| BADLS | -0.04 (0.08) | 0.55 | (-0.19, | 0.10) | -0.05 (0.07) | 0.50 | (-0.19, | 0.09) |
| Age of caregiver (years) | 0.19 (0.05) | <0.01 | (0.09, | 0.29) | 0.17 (0.05) | <0.01 | (0.07, | 0.27) |
| Sex of caregiver (female) | 3.92 (1.48) | 0.009 | (1.00, | 6.84) | 4.45 (1.52) | <0.01 | (1.46, | 7.44) |
| Education (years) | -0.72 (0.90) | 0.43 | (-2.51, | 1.06) | -1.38 (0.96) | 0.15 | (-3.28, | 0.51) |
| Duration of care provided (years) | 0.17 (0.20) | 0.40 | (-0.23, | 0.56) | 0.18 (0.17) | 0.27 | (-0.15, | 0.51) |
| Age of index patient (years) | 0.13 (0.06) | 0.03 | (0.01, | 0.26) | 0.11(0.06) | 0.07 | (-0.01, | 0.23) |
| Sex of index patient (female) | -1.98 (2.21) | 0.37 | (-6.35, | 2.38) | -2.33 (2.13) | 0.28 | (-6.54, | 1.88) |
| Relationship of index patient to caregiver |  |  |  |  |  |  |  |  |
| Parent/parent-in-law | Ref |  |  |  | Ref |  |  |  |
| Grandparent | 5.01 (2.14) | 0.02 | (0.79, | 9.23) | 5.00 (2.00) | 0.01 | (1.04, | 8.95) |
| Spouse/other | 0.65 (2.06) | 0.75 | (-3.42, | 4.73) | 0.36 (2.22) | 0.87 | (-4.03, | 4.75) |

*b, estimated regression coefficient; SE, standard error; CI, confidence interval; ZBI, Zarit Burden Interview*
